# Kinetic Determination of Acarbose and Miglitol in Bulk and Pharmaceutical Formulations Using Alkaline Potassium Permanganate

**Published:** 2007-03

**Authors:** F. A. Ibrahim, F. A. Ali, S. M. Ahmed, M. M. Tolba

**Affiliations:** *Department of Analytical Chemistry, Faculty of Pharmacy, University of Mansoura, Mansoura, Egypt*

**Keywords:** kinetic determination, spectophotometry, acarbose, miglitol, potassium permanganate, pharmaceutical analysis

## Abstract

A simple and sensitive kinetic spectrophotometric method was established for the determination of acarbose and miglitol in bulk and in their pharmaceutical preparations using alkaline potassium permanganate as an oxidizing agent. The method involves determination of acarbose and miglitol by kinetic studies of their oxidation at room temperature for a fixed time of 15 minutes for acarbose and 25 minutes for miglitol. The absorbance of the colored manganate ion was measured at 610 nm. Alternatively, the kinetic decrease in the absorbance of permanganate upon addition of the studied drugs at 525 nm was also used. The absorbance concentration plot was rectilinear over the concentration range of 4-20 and 1-10 μg/ml for acarbose and miglitol, respectively. The detection limits were 0.189 and 0.089 μg/ml at 610 nm and 0.081 and 0.179 μg/ml at 525 nm for acarbose and miglitol respectively. The method was successfully applied for the determination of these drugs in their dosage forms. The results obtained were in good agreement with those obtained with the reference methods.

## INTRODUCTION

Acarbose(O-4,6-dideoxy-4-[[(1S,4R,5S,6S)-4,5,6-tri-hydroxy-3-(hydroxymethyl)-2-cyclohexen-1-yl] amino]-α-D-glucopyranosyl-(1->4)-O-α-D-glucopyranosyl-(1->4)-D-glucose) (1) is an oral alpha-glucosidase inhibitor, especially sucrase. It is given by mouth in the treatment of type 2 diabetes mellitus. It has also been studied for the treatment of reactive hypoglycemia, the dumping syndrome and certain types of hyperlipoproteinaemia (2). Miglitol (2R, 3R, 4R, -5S)-1-(2-hydroxyethyl)-2-(hydroxymethyl)-3,4,5-piperidine-triol) is a desoxynojirimycin derivative, also competitively inhibit glucoamylase and sucrase but has weak effects on pancreatic α-amylase (3).

Some analytical methods have been reported for the determination of the studied drugs. The reported methods for acarbose include Gas Chromatography-Mass spectrophotometry (GC-MS) (4), High Performance Liquid Chromatography (HPLC) (5, 6) and Capillary Electrophoresis (C.E.) (6, 7). As for miglitol, the reported methods were HPLC-MS (8) and C.E. (9).

To the best of our knowledge, no spectrophotometric methods have been reported for the analysis of acarbose and miglitol up till now. The results obtained were promising.

Several analytical techniques such as, spectrophotometry, chromatography, capillary electrophoresis, kinetic fluorimetry, flow injection analysis and chemiluminescence were utilized for selective oxidation and determination of many pharmaceutical compounds in formulations using potassium permanganate as reagent viz, benzenediols and 1,2,4 benzenetriol (10), perchloroethylene (11), arsenic (III) (12), methotrexate (13), propranolol (14), tetracyclines residues (15), psilocin and psilocybin (16), captopril (17), nickel ions (18), cefprozil (19), tramadol hydrochloride (20), ramipril (21), isoxsuprine (22), triprolidine (23), norfloxacin (24), fungicidal ethylenebisdithiocarbamate (25).

The aim of the present work was to study the reaction between the studied drugs with potassium permanganate in alkaline medium kinetically in an attempt to evaluate them in their dosage forms. The proposed method was simple and did not need sophisticated instruments or special skill, sensitive, rapid and readily adaptable to both the bulk drug and dosage forms.

## EXPERIMENTAL

### Reagents

All chemicals used were of analytical reagent grade and the solvents were of spectroscopic grade.

- Potassium permanganate (Merck, Germany), 1 × 10^-2^ M and 7.6 × 10^-3^ M aqueous solutions.
- Sodium hydroxide (BDH, UK), 0.5 M aqueous solution.

### Materials

The different pharmaceutical preparations were purchased from the commercial source in the local market. they include:

- Acarbose was kindly offered from Alkan Pharma S.A.E., Egypt under licence of Bayer-Leverkusen, Germany.
- Miglitol was kindly offered from Sigma Pharmaceutical Industries, Egypt.
- Glucobay 50 tablets, labeled to contain 50 mg acarbose/tablet, Batch # 080. The product of Alkan Pharma S.A.E., Egypt under licence of Bayer- Leverkusen, Germany.
- Glyset tablets, labeled to contain 50mg miglitol/ tablet, Batch # 6191668. The product of Bayer Company, USA.

### Stock solutions

Stock solutions of acarbose and miglitol were prepared by dissolving 100.0 mg of the studied drugs in 100 ml distilled water. Other concentrations were prepared by further dilution with distilled water. These solutions also were found stable for at least three days without alteration when kept in the refrigerator.

### Apparatus

UV-1601, Shimadzu recording spectrophotometer (P/N 206-67001) equipped with kinetic accessory provided with temperature controlled cell (TCC-240A) thermoelectric temperature. Recording range, 0-1; wavelength, 610 and 525 nm; factor 1; number of cell, 1; reaction time (min.) 15, 25 min.; cycle time, 0.1 min.

## GENERAL PROCEDURES

### Construction of the kinetic calibration graphs

Aliquot solutions containing 40-200 μg of standard acarbose and 10-100 μg of miglitol solutions were transferred into a series of 10-ml volumetric flasks. 1 ml of 0.5 M NaOH, followed by 1 or 2.5 ml of 1 × 10^-2^ M potassium permanaganate for acarbose and miglitol, respectively at 610 nm or 0.5 ml of 7.6 × 10^-3^ M for both drugs at 525 nm were added. The mixture was shaken well and completed to volume with distilled water. The increase at 610 nm or the decrease at 525 nm in the absorbance was scanned during 15 and 25 min. for acarbose and miglitol, respectively at ambient temperature (25°C) against an appropriate blank, prepared simultaneously. The reaction order was obtained by plotting log reaction rate (ΔA/Δt) over the specified time period *versus* log concentration of the drug. The calibration graphs and the regression equations were obtained by plotting the absorbance (A) or the difference in absorbance (ΔA) at the specified time *versus* concentration of the drug in μg/ml.

### Procedure for the Determination of the Studied Compounds in Dosage Forms

An accurately weighed quantity of the mixed contents of 10 powdered tablets equivalent to 100.0 mg of the drug was transferred into a 100 ml volumetric flask. The content of the flask was completed to 100 ml with distilled water then sonicated for 15 minutes and filtered. An aliquot of the cited solutions was taken and the above procedure was applied. The nominal content was calculated either from a previously plotted calibration graph or using the regression equation.

## RESULTS AND DISCUSSION

**Figure 1:**
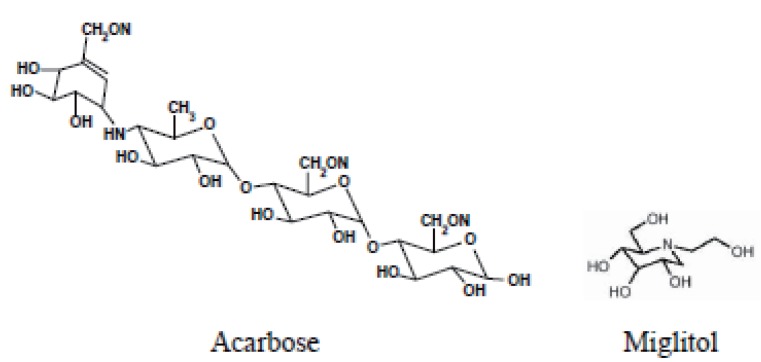
The structure formulae for the studied drugs.

**Figure 2:**
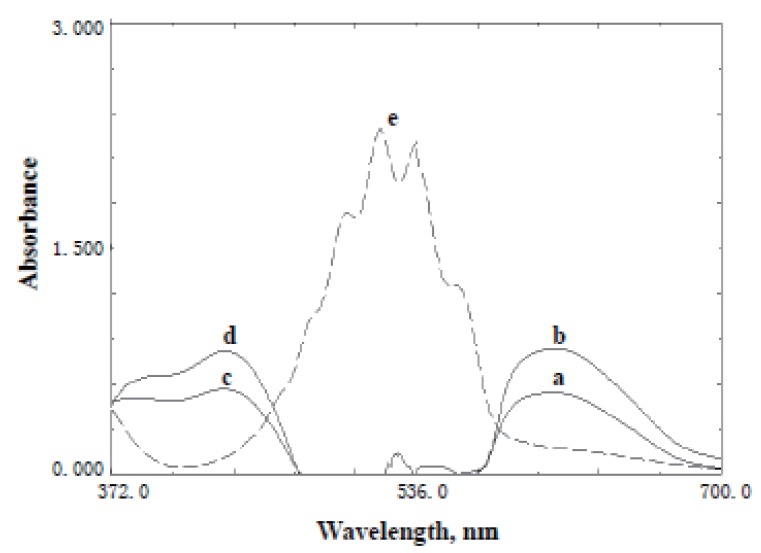
Absorption spectra of the studied drugs after reaction with KMnO_4_/NaOH system. (a, b) The produced manganate ions after the reaction of KMnO_4_ with acarbose (20 μg/ml) or miglitol (10 μg/ml); (c, d) Oxidation products of acarbose and miglitol, respectively; (e) KMnO_4_ (1 × 10^-3^ M).

Oxidation of the studied drugs (Fig. 1) with KMnO_4_ was carried out in presence of NaOH. Trials were made to determine the drug through oxidation with KMnO_4_ in neutral and acidic media, but no apparent reaction products were observed. Potassium permanganate in alkaline medium oxidized the studied drugs and yielded the green color of manganate radical, which absorbs maximally at 610 nm (Fig. 2). The intensity of the color was increased with time, and so, a kinetic method was developed for the determination of these drugs at 610 nm. An alternative kinetic method for the determination of both drugs based upon measuring the decrease in the absorbance of KMnO_4_ at 525 nm was also developed.

Other oxidants were tested to determine the studied drugs, such as 10% H_2_O_2_, potassium persulphate in alkaline medium and potassium periodate in strong acid medium but all failed to give satisfactory results In case of H_2_O_2_ and persulphate, complete decomposition of the drug was observed, as revealed by the absence of any absorbing species. In case of periodate, oxidation of the drug resulted in hypsochromic shift and hypochromic effect, with maximum absorbance at 236 nm and this was in agreement with the reported results of oxidation of amino-alcohol compounds (26).

### Study of Experimental Parameters

The different experimental parameters affecting the formation of the oxidation product were studied. Variables were optimized by changing each in turn, while, keeping all others constant.

**Figure 3:**
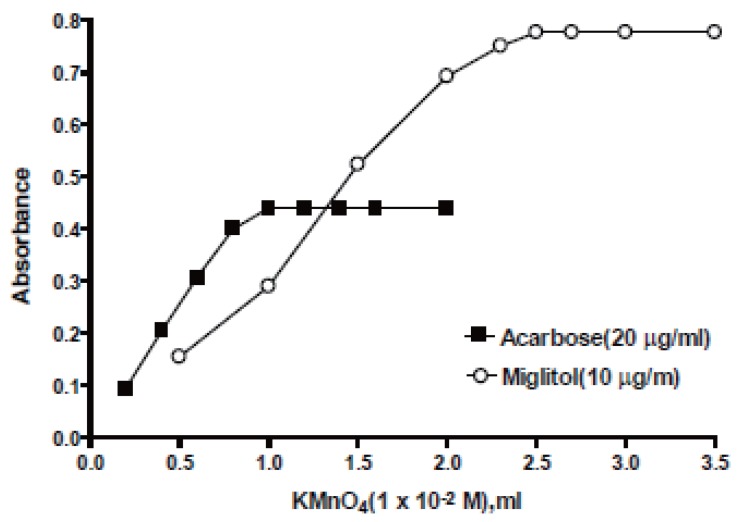
Effect of KMnO_4_ on the absorbance intensity at 610 nm.

**Effect of KMnO_4_ concentration.** The influence of KMnO_4_ concentration on the absorbance of the reaction product was studied using different volumes (0.2-3.5 ml) of 1 × 10^-2^ M KMnO_4_. The reaction rate and hence maximum absorbance increased with increasing KMnO_4_ concentration at 610 nm. It was found that at least 1 or 2.5 ml of 1 × 10^-2^ M KMnO_4_ was adequate for the maximum absorbance of acarbose and miglitol, respectively as shown in Fig. 3, 0.5 ml of 7.6 × 10^-3^ M was sufficient for measuring the decrease in the absorbance at 525 nm for both drugs.

**Figure 4:**
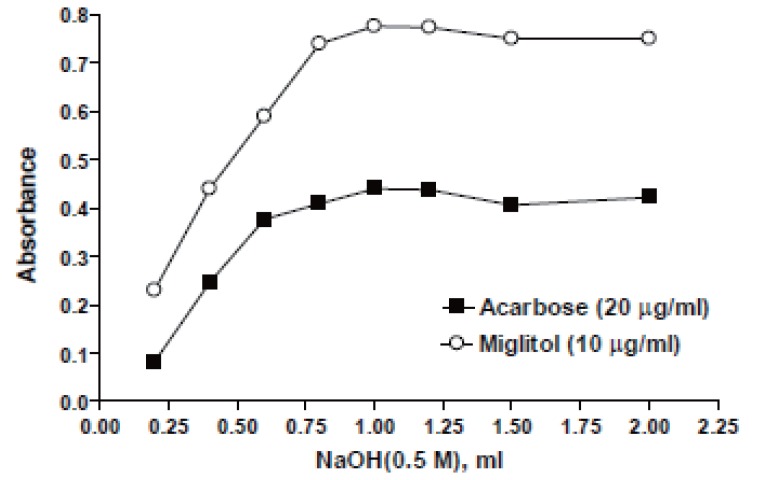
Effect of NaOH on the absorbance intensity at 610 nm.

**Figure 5:**
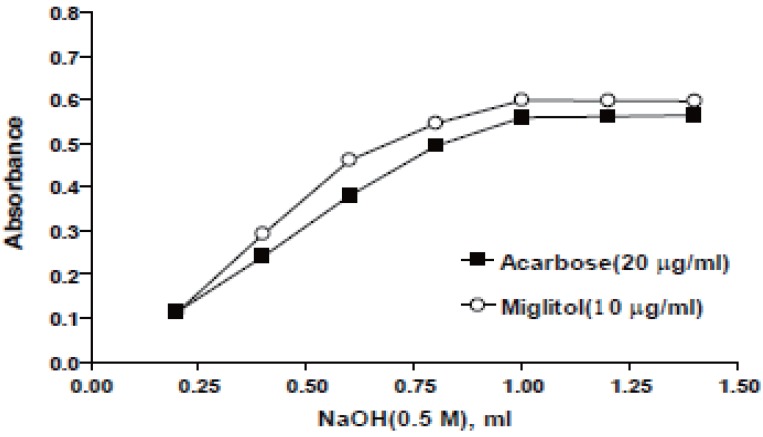
Effect of NaOH on the absorbance intensity at 525 nm.

**Effect of sodium hydroxide concentration.** The influence of the concentration of NaOH on the absorbance of the reaction product was studied using different volumes (0.2-1.4 ml) of 0.5 M NaOH. It was found that increasing the volume of 0.5 M NaOH would increase A or ΔA of the reaction up to 1 ml for both drugs at 610 nm or 525 nm after that NaOH has no effect on the absorbance as shown in Figures 4 & 5.

**Effect of temperature.** The effect of temperature on the reaction rate was studied, it was found that, permanganate was reduced to manganate radical at room temperature (25°C) while at higher temperatures, manganese dioxide was produced. Therefore, room temperature was selected as the optimum temperature.

**Figure 6:**
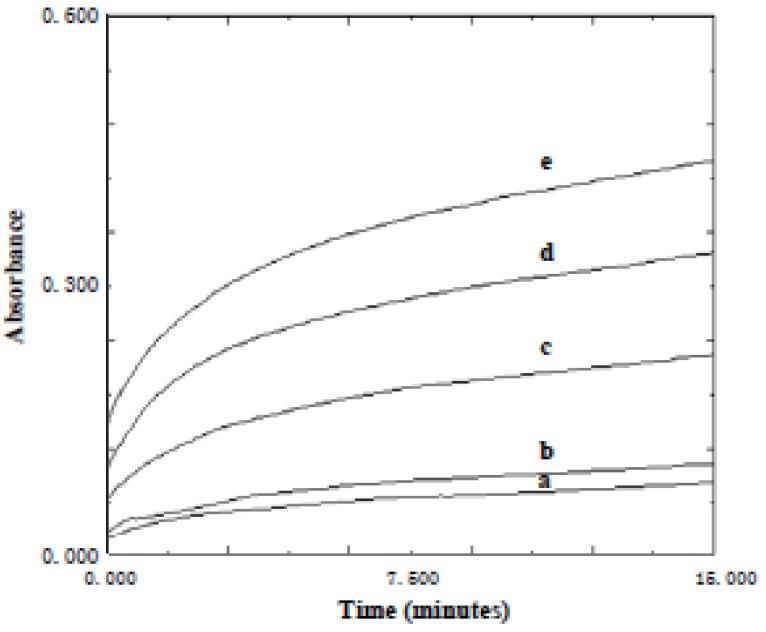
Absorption versus time graphs for the reaction between acarbose and KMnO_4_ at 610 nm. a) 4 μg/ml; b) 5 μg/ml; c) 10 μg/ml; d) 15 μg/ml; e) 20 μg/ml.

**Figure 7:**
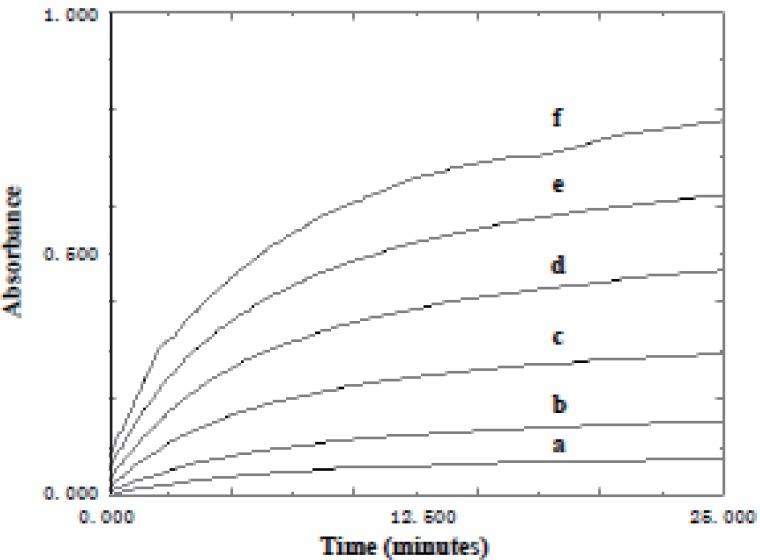
Absorption versus time graphs for the reaction between miglitol and KMnO_4_ at 610 nm. a) 1 μg/ml; b) 2 μg/ml; c) 4 μg/ml; d) 6 μg/ml; e) 8 μg/ml; f) 10 μg/ml.

**Figure 8:**
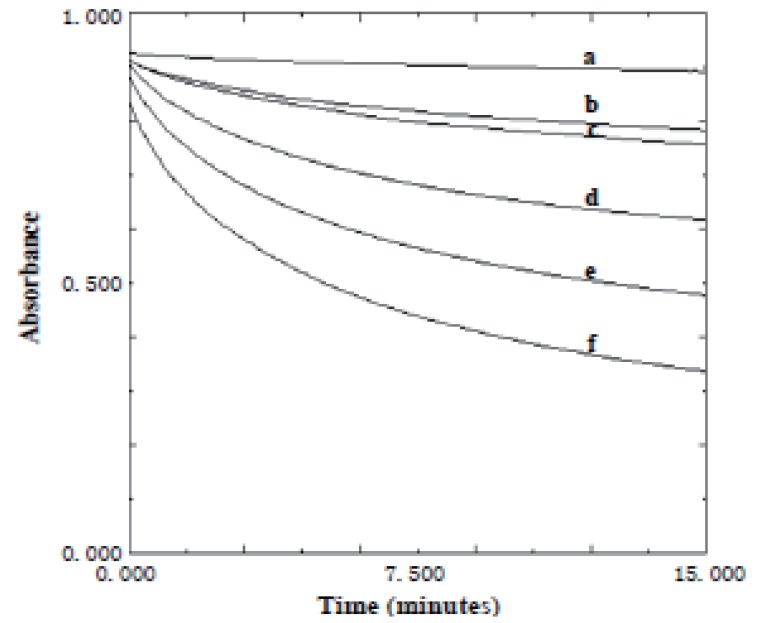
Absorption versus time graphs for the reaction between acarbose and KMnO_4_ at 525 nm. a) Blank; b) 4 μg/ml; c) 5 μg/ml; d) 10 μg/ml; e) 15 μg/ml; f) 20 μg/ml.

**Figure 9:**
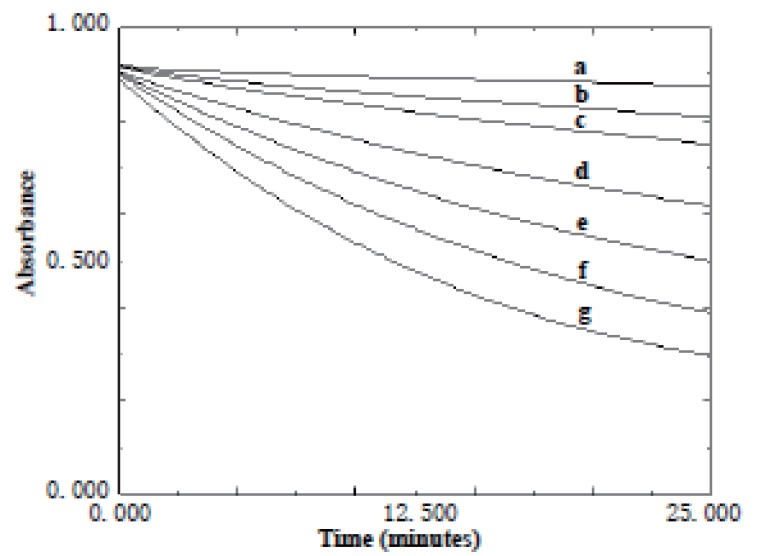
Absorption versus time graphs for the reaction between miglitol and KMnO_4_ at 525 nm. a) Blank; b) 1 μg/ml; c) 2 μg/ml; d) 4 μg/ml; e) 6 μg/ml; f) 8 μg/ml; g) 10 μg/ml.

**Effect of time.** The effect of time on the reaction between KMnO_4_ and the studied drugs was studied. The absorbance of the reaction mixture was increased with time and never reach maximum in a reasonable time. Quantification was therefore made at fixed times of 15 min. for acarbose and 25 min. for miglitol (Figs.6-9).

### Evaluation of the Kinetic Parameters

As mentioned above, the reaction between KMnO_4_ and the studied drugs never reach completion and a decision was made to apply a kinetic method for their determination. Consequently, the order of the reaction and reaction rate constants were determined at 610 and 525 nm.

The rate of the reaction was found to be dependent on acarbose and miglitol concentrations. The rates were followed at room temperature with various concentrations in the range of 4-20 μg/ml for acarbose and 1-10 μg/ml for miglitol keeping KMnO_4_ and NaOH concentrations constant at the recommended levels mentioned above. The reaction rate obeys the following equation:

(a) Rate of the reaction=ΔAΔt=K'drugn

where K` is the pseudo-order rate constant and n is the order of the reaction.

The rate of the reaction may be estimated by the variable time method measurement (27), where A is the absorbance and t is the time in seconds. Taking logarithms of rates and drug concentrations (Table 1), the previous equation is transformed into:

(b) lograte=logΔAΔt=logK'+n logdrug

**Table 1:** Logarithm of the rate for different concentrations of the drugs at room temperature

Compound	Log ΔA/At	Log [drug]	Regression equation	Correlation coefficient	Rate costant (S-1)	Order of reaction (n)

At 610 nm						
Acarbose	-4.138	-5.208	Log rate = 0.865 + 0.962 log C	0.9992	7.328	0.962
	-4.069	-5.111				
	-3.756	-4.810				
	-3.586	-4.634				
	-3.483	-4.509				
Miglitol						
	-4.309	-5.316	Log rate = 0.916 +0.982 log C	0.9999	8.241	0.982
	-4.010	-5.015				
	-3.714	-4.714				
	-3.537	-4.538				
	-3.420	-4.413				
	-3.329	-4.316				
At 525 nm						
Acarbose	-3.974	-5.208	Log rate = 1.243 +1.001 log C	0.9993	17.49	1.001
	-3.872	-5.111				
	-3.563	-4.810				
	-3.380	-4.634				
	-3.285	-4.509				
Miglitol	-4.359	-5.316	Log rate = 0.662 + 0.946 log C	0.9999	4.591	0.946
	-4.090	-5.015				
	-3.801	-4.714				
	-3.630	-4.538				
	-3.507	-4.413				
	-3.420	-4.316				

Plot of log reaction rate versus log drug concentration at 610 nm and 525 nm gave the regression equation, correlation coefficient, pseudo-order rate constant and order of the reaction which are indicated in Table 1. These results indicate that the reaction is pseudo first order reaction in the drug concentration.

### Selection of the best kinetic method

Several kinetic techniques were adopted for the selection of the best method. Rate constant, fixed absorbance and fixed time methods (28, 29) were tried and the most suitable analytical method was selected taking into account the applicability, the sensitivity, i.e. the slope of the calibration graph and the correlation coefficient (r).

Rate constant method. Graphs of log absorbance versus time for acarbose and miglitol concentration in the range of 6.20 × 10^-6^ - 3.10 × 10^-5^ M and 4.83 × 10^-6^ - 4.83 × 10^-5^ M, respectively were plotted and all appeared to be rectilinear. Pseudo-first order rate constants (K`) corresponding to different drug concentrations (C) were calculated from the slopes multiplied by –2.303 and are presented in Table 2.

**Table 2:** Application of the rate constant method in the quantification of the studied drugs with KMnO_4_

Compound	[drug]	K`/S^-1^	
		at 610 nm	at 525 nm

Acarbose	6.195 × 10^-6^		-9.509 × 10^-4^
	7.744 × 10^-6^	-1.474 × 10^-3^	-9.230 × 10^-4^
	1.549 × 10^-5^	-1.350 × 10^-3^	-9.198 × 10^-4^
	2.323 × 10^-5^	-1.297 × 10^-3^	-8.338 × 10^-4^
	3.098 × 10^-5^	-1.188 × 10^-3^	-7.375 × 10^-4^
Miglitol	4.826 × 10^-6^	-5.021 × 10^-4^	-1.005 × 10^-3^
	9.652 × 10^-6^	-4.982 × 10^-4^	
	1.931 × 10^-5^	-4.737 × 10^-4^	-9.557 × 10^-4^
	2.896 × 10^-5^	-4.506 × 10^-4^	-9.343 × 10^-4^
	3.861 × 10^-5^		-9.112 × 10^-4^
	4.826 × 10^-5^	-4.291 × 10^-4^	-8.245 × 10^-4^

K`, the pseudo first order rate constant.

Regression of (C) versus K` gave equations:

- At 610 nm
  K` = -1.56 × 10^-3^ + 11.76 C (r=0.9900) for acarbose
  K` = -5.10 × 10^-4^ + 1.78 C (r=0.9850) for miglitol
- At 525 nm
  K`= -1.01 × 10^-3^ + 8.17C (r=0.9590) for acarbose
  K`= -1.03 × 10^-3^ + 3.78 C (r=0.9570) for miglitol

where C is the molar concentration of the drugs.

Fixed absorbance method. Reaction times required to reach specific absorbance of redox reaction for different concentrations of acarbose and miglitol 1.55 × 10^-5^ – 3.10 × 10^-5^ M and 1.93 × 10^-5^ – 4.83 × 10^-5^ M, respectively were recorded. A preselected value of the absorbance (0.2) for acarbose and miglitol was fixed and the time was measured in seconds. The reciprocal of time (1/t) versus the initial concentration of drug was plotted. Table 3 and the following equations of the calibration graphs were obtained:

- At 610 nm
  1/t = -2.92 × 10^-2^ + 1847.57 C (r=0.9650) for acarbose
  1/t = -5.89 × 10^-3^ + 402.04 C (r=0.9960) for miglitol
- At 525 nm
  1/t = -1.38 × 10^-2^ + 1010.16 C (r=0.9750) for acarbose
  1/t = -7.92 × 10^-4^ + 87.92 C (r=0.9960) for miglitol

where C is the molar concentration of the drugs.

**Table 3:** Application of the fixed absorbance method in the quantification of the studied drugs with KMnO_4_

Compound	[drug]	1/t (sec.^-1^)	
		At 610 nm	At 525 nm

Acarbose	1.549 × 10^-5^	1.684 × 10^-3^	2.874 × 10^-3^
	2.323 × 10^-5^	9.259 × 10^-3^	7.576 × 10^-3^
	3.098 × 10^-5^	3.030 × 10^-2^	1.852 × 10^-2^
Miglitol	1.931 × 10^-5^	2.252 × 10^-3^	9.803 × 10^-4^
	2.896 × 10^-5^	5.376 × 10^-3^	1.701 × 10^-3^
	3.861 × 10^-5^	9.259 × 10^-3^	2.488 × 10^-3^
	4.826 × 10^-5^	1.389 × 10^-2^	3.546 × 10^-3^

Fixed time method. At a preselected fixed time, which was accurately determined, the absorbance was measured. Calibration graphs of the absorbance versus initial concentrations of acarbose and miglitol at fixed times of 15 and 25 min., respectively were established with the regression equations and correlation coefficients assembled in Table 4.

**Table 4:** Application of the fixed time method in the quantification of the studied drugs with KMnO_4_

Compound	Time (min.)	Regression Equations	Correlation Coefficient

At 610 nm			
Acarbose	2.5	A= -0.0148 + 0 .0081C	r = 0.9980
	5	A= -0.0133 + 0.0180 C	r = 0.9997
	10	A= -0.0111 + 0.0207 C	r = 0.9998
	15	A= -0.0073 + 0.0224C	r = 0.9999
Miglitol	5	A= -0.0105 + 0.0459 C	r = 0.9996
	10	A= -0.0094 + 0.0615C	r = 0.9997
	15	A= -0.0080 + 0.0694 C	r = 0.9998
	20	A= -0.0053 + 0.0741 C	r = 0.9998
	25	A= -0.0038 + 0.0780 C	r = 0.9999
At 525 nm			
Acarbose	2.5	A= -0.0170 + 0.0160 C	r = 0.9970
	5	A= -0.0173 + 0.0209 C	r = 0.9994
	10	A= -0.0094 + 0.0256 C	r = 0.9999
	15	A= -0.0043 + 0.0280 C	r = 0.9999
Miglitol	5	A= -0.0067 + 0.0212 C	r = 0.9980
	10	A= -0.0095 + 0.0360 C	r = 0.9993
	15	A= 0.0002 + 0.0451 C	r = 0.9995
	20	A= 0.0045 + 0.0534 C	r = 0.9996
	25	A= 0.0098 + 0.0594C	r = 0.9998

It is clear that the slope increases with time and the most acceptable values of the correlation coefficient (r) was chosen as the most suitable time interval for the measurement.

As a conclusion, the fixed time method was chosen for quantification because it gave the best correlation coefficient in a reasonable time.

### Quantification

After optimizing the reaction conditions, the fixed time method was applied to the kinetic determination of 4-20 μg/ml of acarbose and 1-10 μg/ml of miglitol in raw materials.

Analysis of the data gave the following regression equations:

- At 610 nm
  A= -7.332 × 10^-3^ + 2.243 × 10^-2^ C (r=0.9999) for acarbose
  A= -3.782 × 10^-3^ + 7.797 × 10^-2^ C (r=0.9999) for miglitol
- At 525 nm
  A= -4.250 × 10^-3^ + 2.799 × 10^-2^ C (r=0.9999) for acarbose
  A= 9.787 × 10^-3^ + 5.939 × 10^-2^ C (r=0.9998) for miglitol

where A is the absorbance and C is the concentration in μg/ml.

Statistical evaluation of the regression line gave the values of S_y/x_, S_a_, S_b_ which are indicated with the detection limits, quantification limits, % RSD and % Er. in Table 5. These small values point out to the high precision of the proposed methods.

**Table 5:** Collective parameters for the determination of the studied drugs by the proposed methods

Compounds	At 525 nm		At 610 nm	
	Miglitol	Acarbose	Miglitol	Acarbose

Concentration range (μg/ml)	1-10	4-20	1-10	4-20
Detection limit (μg/ml)	0.179	0.081	0.089	0.189
Quantification limit (μg/ml)	0.596	0.269	0.297	0.630
S_y/x_	4.220 × 10^-3^	1.210 × 10^-3^	2.762 × 10^-3^	2.269 × 10^-3^
S_a_	3.538 × 10^-3^	7.538 × 10^-4^	2.316 × 10^-3^	1. 413 × 10^-3^
S_b_	5.411 × 10^-4^	8.950 × 10^-5^	3.541 × 10^-4^	1.678 × 10^-4^
% RSD	1.269	0.719	1.186	1.095
% Er	0.568	0.322	0.484	0.490

S_y/x_, Standard deviation of the residual; S_a_, Standard deviation of the intercept; S_b_, Standard deviation of the slope; % RSD, Relative standard deviation; % Er, Percentage error.

Statistical analysis of the results obtained by both the proposed and reference methods (30, 31) revealed no significant difference regarding the accuracy and precision as indicated by the Student t-test and F test (32), as shown in Table 6. The reference method (30) for acarbose involved an optical rotation method given by Alkan Pharma S.A.E., Egypt under licence of Bayer- Leverkusen, Germany. While, the reference method (31) for miglitol was an HPLC method using acetonitrile and 25 mM sodium dibasic phosphate as mobile phase and detection at 205 nm. The data was provided by Sigma Pharmaceutical Industries, Egypt.

**Table 6:** Application of the proposed methods for the determination of the studied drugs in their raw materials

Compound	Proposed methods			Reference methods (30, 31)	
	Amount taken (μg/ml)	Amount found (μg/ml)	Recovery (%)	Amount taken (μg/ml)	Recovery (%)

At 610 nm					
Acarbose	4.0	3.978	99.45		
	5.0	4.919	98.38		
	10.0	10.135	101.35		
	15.0	15.039	100.26		
	20.0	19.926	99.63		
x ± S.D.			99.81 ± 1.093	99.20 ± 0.400	
t			0.906	(2.447)^a^	
F			7.47	(19.25)	
Miglitol	1.0	1.021	102.10		
	2.0	2.015	100.75	60.0	101.00
	4.0	3.937	98.43	80.0	98.50
	6.0	6.019	100.32	100.0	100.61
	8.0	8.000	100.00		
	10.0	10.009	100.09		
x ± S.D.			100.28 ± 1.189	100.04 ± 1.350	
t			0.274	(2.365)^a^	
F			1.29	(19.30)	
At 525 nm					
Acarbose	4.0	4.039	100.98		
	5.0	4.950	99.00		
	100	10.027	100.27		
	15.0	14.971	99.81		
	20.0	20.013	100.06		
x ± S.D.			100.02 ± 0.719	99.20 ± 0.400	
t			1.779	(2.447)^a^	
F			3.23	(19.25)	
Miglitol	2.0	1.967	98.35		
	4.0	4.045	101.13	60.0	101.00
	6.0	6.067	101.12	80.0	98.50
	8.0	8.052	100.65	100.0	100.61
	10.0	9.911	99.11		
x ± S.D.			100.07 ± 1.270	100.04 ± 1.350	
t			0.032	(2.447)^a^	
F			1.13	(19.25)	

Each result is the average of three separate determinations.

a Figures in parentheses are the tabulated t and F values, respectively at *p*=0.05 (32).

The proposed methods were successfully applied for the determination of the studied drugs in their different dosage forms. The results obtained were in a good agreement with the reference methods (30, 31), as shown in Table 7.

**Table 7:** Application of the proposed methods for the determination of the studied drugs in pharmaceutical preparations

Pharmaceutical preparations	Proposed methods			Reference methods	
	Amount taken (μg/ml)	Amount found (μg/ml)	Recovery (%)	Taken (μg/ml)	Recovery (%)

At 610 nm					
Glucobay 50 tablets^a^	5.0	5.008	100.16		
(Acarbose, 50 mg/tablet)	10.0	10.091	100.91		
	15.0	15.218	101.45		
x ± S.D.			100.84 ± 0.648	100.400 ± 0.400	
t			1.002	(2.776)^c^	
F			2.62	(19.00)	
Glyset 50 mg tablets^b^	2.0	1.978	98.90	60.0	97.26
(Miglitol, 50 mg/tablet)	4.0	4.025	100.63	80.0	98.71
	8.0	8.099	101.24	100.0	99.66
x ± S.D.			100.26 ± 1.214	98.54 ± 1.208	
t			1.740	(2.776)^c^	
F			1.01	(19.00)	
At 525 nm					
Glucobay 50 tablets	4.0	4.044	101.10		
(Acarbose, 50 mg/tablet)	10.0	10.062	100.62		
	20.0	20.220	101.10		
x ± S.D.			100.94 ± 0.277	100.400 ± 0.400	
t			1.923	(2.776)^c^	
F			2.086	(19.00)	
Glyset 50 mg tablets	2.0	1.987	99.35	60.0	97.26
(Miglitol, 50 mg/tablet)	4.0	4.070	101.75	80.0	98.71
	8.0	8.036	100.45	100.0	99.66
x ± S.D.			100.52 ± 1.201	98.54 ± 1.208	
t			2.014	(2.776)^c^	
F			1.01	(19.00)	

Each result is the average of three separate determinations.

a Product of Alkan Pharma S.A.E., Egypt under licence of Bayer-Leverkusen, Germany, Egypt;

b Product of Sigma Pharma-ceutical industries, Egypt;

c Figures in parentheses are the tabulated t and F values, respectively at *p*=0.05 (32).

### Mechanism of the reaction

The data used in the optimization of KMnO_4_ concentration and the data of the calibration graphs were used to calculate the stoichiometry of the reaction adopting the limiting logarithmic method (33).

**Figure 10:**
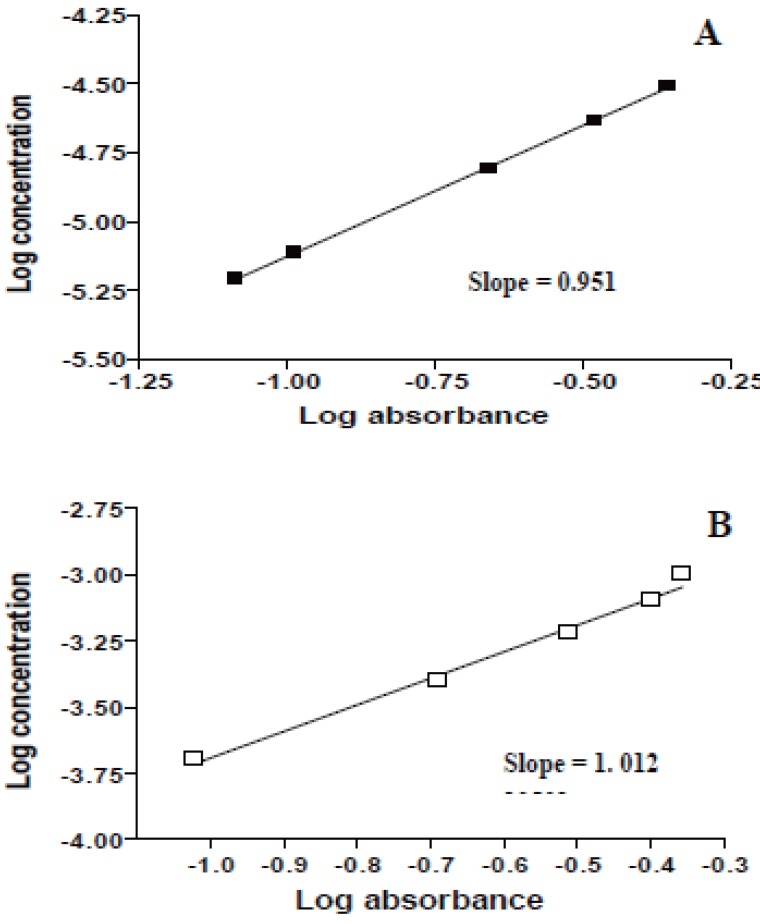
Stoichiometry of the reaction between acarbose and KMnO_4_ adopting limiting logarithmic method (33). A) Variable concentrations of acarbose at constant KMnO_4_ concentration; B) Variable concentrations of KMnO_4_ at constant acarbose concentration.

**Figure 11:**
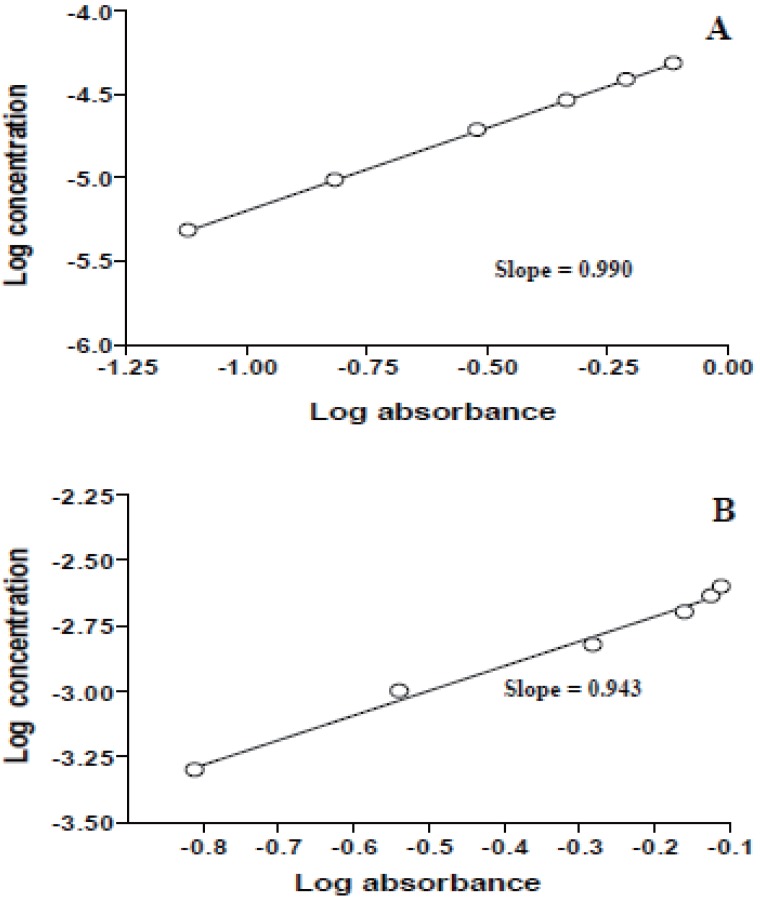
Stoichiometry of the reaction between miglitol and KMnO_4_ adopting limiting logarithmic method (33). A) Variable concentrations of miglitol at constant KMnO_4_ concentration; B) Variable concentrations of KMnO_4_ at constant miglitol concentration.

The ratio of the reaction between acarbose or miglitol and KMnO_4_ in alkaline medium was calculated by dividing the slope of KMnO_4_ curve over the slope of the drug curve (Figs. 10 & 11). It was found that, the ratios were 1.012: 0.951 for acarbose and 0.943: 0.990 for miglitol pointing out to a ratio of 1:1 (KMnO_4_ to drug).

**Figure 12:**
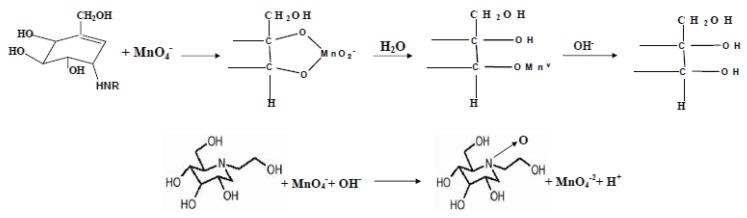
The proposed pathway for the reaction between the studied drugs and potassium permanganate in alkaline medium.

Based on the obtained molar reactivity, the reaction pathway is proposed to proceed as in Figure 12.

## CONCLUSION

The proposed methods are simple, accurate, precise, sensitive, rapid, low cost and selective compared to the reference methods (30, 31).

Furthermore, the proposed methods don’t require elaboration of procedures, which are usually associated with chromatographic methods. The proposed methods were applied successfully for determination of the studied compounds in raw material as well as in different dosage forms. The only limitation for this method, if used in other pharmaceutical preparations containing antioxidant which will cause interference and this can be solved by using suitable solvent extraction.

From the above study some specific advantages in the application of kinetic methods can be expected (35):

- Selectivity due to the measurement of the evolution of the absorbance with the time of reaction instead of the measure of a concerete absorbance value.
- Possibility of no interference of other absorbent active compounds present in the commercial product, if they are exhibiting stability in the chemical reaction conditions established for the proposed kinetic method.
- Possibility of no interference of the colored and /or turbidity back ground of the sample.
